# Prognostic Value of Systemic Immune-Inflammation Index for Early Mortality After Decompressive Craniectomy in Malignant Middle Cerebral Artery Infarction

**DOI:** 10.3390/brainsci16070666

**Published:** 2026-06-25

**Authors:** Yasin Taşkın, Özgür Demir, Veysel Kıyak, Mustafa Arslan, Övgü Can Ünal, Yunus Emre Kuyucu

**Affiliations:** 1 Department of Neurosurgery, Tokat Gaziosmanpaşa University, 60030 Tokat, Turkey; cerendemir40@gmail.com (Ö.D.); vyslkyk86@gmail.com (V.K.); can.ovgucan68@gmail.com (Ö.C.Ü.); 2 Department of Neurosurgery, Gaziantep City Hospital, 27470 Gaziantep, Turkey; mustafaarslan_60@hotmail.com.tr; 3 Department of Biostatistics, Faculty of Medicine, Tokat Gaziosmanpaşa University, 60030 Tokat, Turkey; kuyucuemre@hotmail.com

**Keywords:** malignant MCA infarction, decompressive craniectomy, systemic immune-inflammation index, neutrophil-lymphocyte ratio, mortality

## Abstract

**Highlights:**

**What are the main findings?**

- Systemic immune–inflammation index (SII) was an independent predictor of in-hospital mortality after decompressive craniectomy for malignant middle cerebral artery infarction.
- SII demonstrated superior prognostic performance compared with neutrophil-to-lymphocyte ratio (NLR), achieving the highest predictive accuracy in ROC analysis (AUC = 0.833).
- Advanced age, elevated serum creatinine, hypertension, and preoperative anticoagulant therapy were independently associated with increased in-hospital mortality.

**What are the implications of the main findings?**

- SII may serve as a simple, inexpensive, and readily available biomarker for early mortality risk stratification in patients undergoing decompressive craniectomy for malignant MCA infarction.
- Integration of SII into preoperative assessment may improve clinical decision-making, postoperative monitoring, and identification of high-risk patients who may require intensified management.

**Abstract:**

**Objective:** Malignant middle cerebral artery infarction is associated with high mortality despite decompressive craniectomy. Reliable biomarkers predicting early outcome remain limited. The aim of this study was to evaluate the prognostic significance of inflammatory biomarkers, particularly the systemic immune–inflammation index (SII), for predicting in-hospital mortality in patients undergoing decompressive craniectomy for malignant middle cerebral artery infarction. **Methods:** This retrospective study included 31 patients who underwent decompressive craniectomy for malignant MCA infarction between 2014 and 2024. Demographic, clinical, radiological, and laboratory variables were analyzed. **Results:** Overall in-hospital mortality was 61.3%. Non-survivors had significantly higher SII, neutrophil count, neutrophil-to-lymphocyte ratio, serum creatinine, and higher prevalence of hypertension and anticoagulant therapy. ROC analysis showed that SII had the highest predictive performance (AUC = 0.833). Multivariate analysis identified age, serum creatinine, NLR, SII, hypertension, and anticoagulant therapy as independent predictors of mortality. Patients aged ≥65 years had significantly higher in-hospital mortality than younger patients. **Conclusions:** Elevated SII is a strong independent predictor of early mortality after decompressive craniectomy and may serve as a simple and clinically applicable biomarker for risk stratification.

## 1. Introduction

Malignant middle cerebral artery (MCA) infarction represents one of the most devastating subtypes of ischemic stroke, characterized by extensive cerebral edema, increased intracranial pressure, and a high risk of fatal brain herniation despite optimal medical therapy [1]. Decompressive craniectomy (DC) has been shown to significantly reduce mortality and improve functional outcomes in selected patients by alleviating intracranial hypertension and preventing secondary brain injury [2,3]. However, mortality rates following DC remain substantial, and reliable predictors of early outcome are still needed to optimize patient selection and perioperative management [4].

Increasing evidence suggests that systemic inflammatory responses play a crucial role in the pathophysiology of ischemic brain injury and secondary edema formation [5]. Following cerebral ischemia, activation of neutrophils, disruption of the blood–brain barrier, and release of pro-inflammatory mediators contribute to infarct progression and worsening neurological outcomes [6]. Therefore, easily accessible inflammatory biomarkers have attracted considerable attention as potential prognostic indicators in stroke patients.

Among these markers, the neutrophil-to-lymphocyte ratio (NLR) has emerged as a simple and cost-effective indicator reflecting the balance between innate and adaptive immune responses. Previous studies have demonstrated that elevated NLR values are associated with increased infarct volume, poor functional recovery, and higher mortality in patients with acute ischemic stroke [7,8]. More recently, the systemic immune–inflammation index (SII), calculated using neutrophil, lymphocyte, and platelet counts, has been proposed as a more comprehensive marker of inflammatory burden and thrombo-inflammatory activity [9]. Elevated SII levels have been reported to predict unfavorable outcomes in various cardiovascular and cerebrovascular conditions, including ischemic stroke [10].

Despite these findings, the prognostic significance of inflammatory indices in patients undergoing decompressive craniectomy for malignant MCA infarction remains insufficiently investigated. In particular, the independent predictive value of SII and its potential superiority over conventional inflammatory markers such as NLR in this specific high-risk population have not been fully clarified.

Therefore, the aim of the present study was to evaluate the prognostic value of inflammatory biomarkers, particularly the systemic immune–inflammation index, in predicting in-hospital mortality among patients undergoing decompressive craniectomy for malignant MCA infarction.

## 2. Materials and Methods

### 2.1. Study Design and Ethical Approval

This retrospective observational study was conducted at a tertiary university hospital between January 2014 and December 2024. Ethical approval was obtained from the local University Non-Interventional Scientific Research Ethics Committee (approval number: 25-MOBAEK-010; date: 21 January 2025), and the study was performed in accordance with the Declaration of Helsinki.

### 2.2. Patient Population

**Figure 1:**
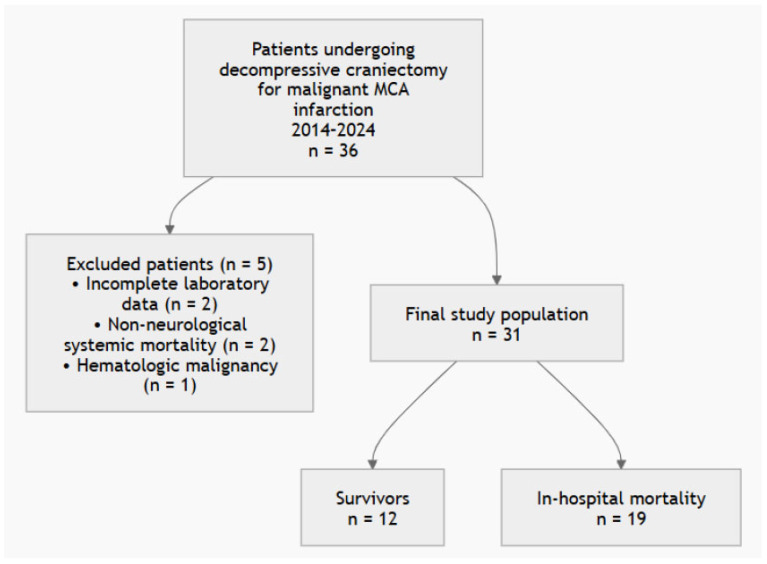
Study flow diagram demonstrating patient selection and exclusion criteria. Thirty-six patients who underwent decompressive craniectomy for malignant middle cerebral artery infarction between 2014 and 2024 were screened. After exclusion of five patients, 31 patients were included in the final analysis.

During the study period, 36 consecutive patients who underwent decompressive craniectomy for malignant middle cerebral artery infarction were screened. Five patients were excluded due to incomplete laboratory data at admission (n = 2), mortality related to non-neurological systemic causes such as sepsis or cardiac complications (n = 2), and a history of hematologic malignancy potentially affecting inflammatory parameters (n = 1). Consequently, 31 patients were included in the final analysis (Figure 1).

Inclusion criteria were age ≥ 18 years, radiologically confirmed MCA infarction on cranial CT and/or MRI, performance of decompressive craniectomy due to neurological deterioration or life-threatening mass effect, and availability of complete clinical and laboratory data at admission, whereas exclusion criteria comprised incomplete medical records, mortality unrelated to the primary neurological pathology or surgical intervention, hematologic malignancy or severe immunodeficiency, active systemic infection at admission, and previous intracranial surgery or major head trauma within the preceding three months.

### 2.3. Surgical Technique and Postoperative Management

All patients underwent decompressive craniectomy using a standardized technique consisting of wide fronto-temporo-parietal craniectomy followed by expansile duraplasty. Surgical indication was based on neurological deterioration, radiological evidence of significant mass effect or midline shift, and multidisciplinary decision-making between neurology and neurosurgery teams.

Postoperative management protocols including osmotherapy, ventilatory support, intracranial pressure control, and hemodynamic monitoring were applied according to institutional neurocritical care guidelines.

### 2.4. Data Collection

Demographic, clinical, radiological, laboratory, and outcome data were retrospectively obtained from electronic hospital records. Clinical variables included Glasgow Coma Scale score at admission, presence of anisocoria, infarct laterality, degree of midline shift, time to surgery, preoperative treatment modalities (mechanical thrombectomy, intravenous tissue plasminogen activator, or medical therapy), anticoagulant therapy, hemorrhagic transformation, and accompanying systemic comorbidities.

Hypertension was defined as a documented history of hypertension or regular use of antihypertensive medication prior to admission. Diabetes mellitus was defined as a previous diagnosis requiring antidiabetic treatment or fasting plasma glucose ≥126 mg/dL at admission. Atrial fibrillation was considered present if previously diagnosed or detected on admission electrocardiography. Coronary artery disease was defined as a history of myocardial infarction, coronary revascularization, or angiographically documented coronary stenosis. Hyperlipidemia was defined as prior lipid-lowering therapy or total cholesterol ≥ 200 mg/dL. Previous stroke was defined as a documented history of ischemic or hemorrhagic cerebrovascular disease.

Radiological evaluations were independently performed by two experienced neurosurgeons blinded to clinical outcomes, and discrepancies were resolved by consensus.

### 2.5. Laboratory Measurements

Venous blood samples were obtained within the first hour of emergency department admission. Laboratory parameters included hemoglobin, neutrophil count, lymphocyte count, and platelet count. From these measurements, inflammatory indices were calculated as neutrophil-to-lymphocyte ratio (NLR) = neutrophil count/lymphocyte count and systemic immune–inflammation index (SII) = (neutrophil × platelet)/lymphocyte. SII values were calculated individually for each patient using admission laboratory parameters, and group-level SII values represent the mean ± standard deviation of these individual calculations rather than values derived from mean neutrophil, lymphocyte, and platelet counts.

### 2.6. Outcome Measures

The primary endpoint was in-hospital mortality, defined as death occurring during the same hospitalization period following decompressive craniectomy.

### 2.7. Statistical Analysis

Statistical analyses were performed using IBM SPSS Statistics version 25.0 (IBM Corp., Armonk, NY, USA). Continuous variables were expressed as mean ± standard deviation and categorical variables as number (percentage). Distribution normality was assessed using the Kolmogorov–Smirnov test. Group comparisons were performed using Student’s *t*-test or the Mann–Whitney U test for continuous variables and the Chi-square test or Fisher’s exact test for categorical variables.

**Figure 2:**
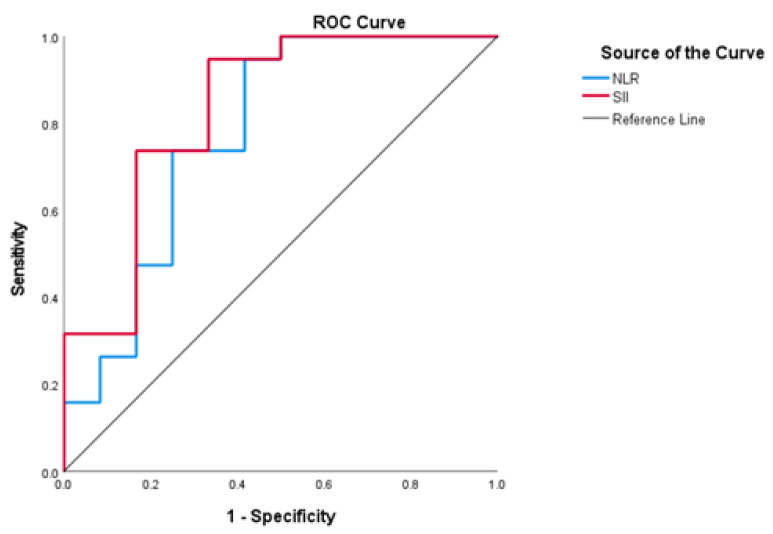
Receiver operating characteristic (ROC) curve analysis of inflammatory biomarkers for prediction of in-hospital mortality following decompressive craniectomy in patients with malignant middle cerebral artery infarction. The systemic immune–inflammation index (SII) demonstrated higher predictive performance compared with neutrophil-to-lymphocyte ratio (NLR).

Receiver operating characteristic (ROC) curve analysis was conducted to evaluate the predictive performance of inflammatory markers for in-hospital mortality. Optimal cut-off values were determined using the Youden index (J = sensitivity + specificity − 1), which identifies the threshold that maximizes the combined sensitivity and specificity of a diagnostic test (Figure 2). Variables with *p* < 0.10 in univariate analysis were entered into a multivariate logistic regression model to determine independent predictors of mortality. A two-tailed *p* value < 0.05 was considered statistically significant.

## 3. Results

A total of 31 patients who underwent decompressive craniectomy for malignant middle cerebral artery infarction were included in the study. Of these, 19 patients (61.3%) died during hospitalization.

### 3.1. Baseline Characteristics According to Age Groups

Baseline demographic, clinical, laboratory, and treatment characteristics according to age groups are presented in Table 1. Patients aged ≥65 years had significantly higher systemic immune–inflammation index (SII) values compared with younger patients (3300 ± 1850 vs. 1900 ± 900, *p* = 0.002). Moreover, in-hospital mortality was significantly higher in the older age group (76.0% vs. 30.0%, *p* = 0.003). No significant differences were observed between the groups regarding neurological status at admission, radiological findings, comorbidities, or treatment variables.

### 3.2. Comparison Between Survivors and Non-Survivors

Comparisons of demographic, clinical, laboratory, inflammatory, and treatment parameters between survivors and non-survivors are summarized in Table 2. Patients who died during hospitalization were significantly older than survivors (71.8 ± 8.9 vs. 58.6 ± 11.2 years, *p* = 0.002). In addition, non-survivors had significantly higher serum creatinine levels (*p* = 0.03), neutrophil counts (*p* = 0.018), neutrophil-to-lymphocyte ratio (*p* = 0.013), and SII values (*p* = 0.002), while lymphocyte counts were significantly lower (*p* = 0.033). Hypertension (*p* = 0.02) and preoperative anticoagulant therapy (*p* = 0.04) were also more common among deceased patients. Other clinical and radiological parameters were not significantly associated with mortality.

### 3.3. ROC Analysis for Prediction of In-Hospital Mortality

Receiver operating characteristic (ROC) analysis of laboratory and inflammatory markers is shown in Table 3 and Figure 2. Among the evaluated parameters, SII demonstrated the highest predictive performance for in-hospital mortality (AUC = 0.833, 95% CI: 0.67–0.99, *p* = 0.002), followed by neutrophil-to-lymphocyte ratio (AUC = 0.776, *p* = 0.013) and neutrophil count (AUC = 0.750, *p* = 0.018). The optimal cut-off value for SII was 2850, yielding a sensitivity of 84% and specificity of 75%. In contrast, lymphocyte count showed poor discriminatory ability (AUC = 0.268).

### 3.4. Multivariate Logistic Regression Analysis

**Figure 3:**
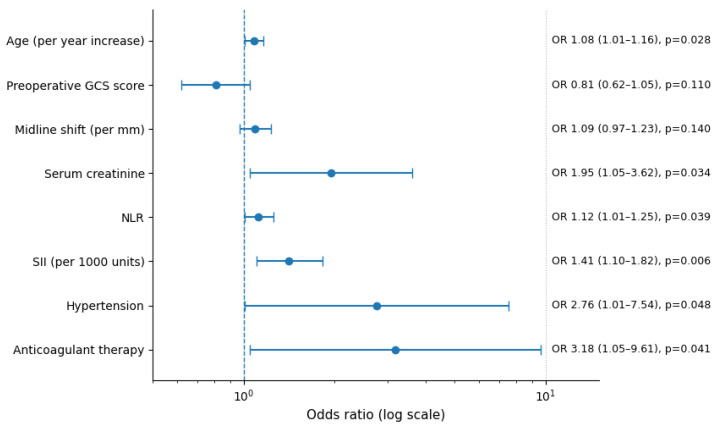
Forest plot of independent predictors of in-hospital mortality following decompressive craniectomy in patients with malignant middle cerebral artery infarction. Odds ratios (ORs) with 95% confidence intervals are presented based on multivariate logistic regression analysis.

Variables with *p* < 0.10 in univariate analysis were included in the multivariate logistic regression model (Table 4). Increasing age (OR = 1.08 per year, *p* = 0.028), elevated serum creatinine (OR = 1.95, *p* = 0.034), higher neutrophil-to-lymphocyte ratio (OR = 1.12, *p* = 0.039), increased SII (OR = 1.41 per 1000 units, *p* = 0.006), hypertension (OR = 2.76, *p* = 0.048), and anticoagulant therapy (OR = 3.18, *p* = 0.041) were identified as independent predictors of in-hospital mortality. The relative contribution and effect sizes of these independent predictors are illustrated in Figure 3.

## 4. Discussion

In the present study, we investigated the prognostic significance of inflammatory biomarkers in patients undergoing decompressive craniectomy for malignant middle cerebral artery infarction and demonstrated that elevated systemic immune–inflammation index (SII), higher neutrophil-to-lymphocyte ratio (NLR), advanced age, increased serum creatinine, hypertension, and preoperative anticoagulant therapy were independently associated with increased in-hospital mortality. These findings highlight the complex interaction between systemic inflammatory burden, baseline clinical characteristics, and postoperative survival in this high-risk population.

Malignant MCA infarction is associated with substantial mortality despite aggressive medical and surgical management. Randomized trials and pooled analyses have consistently shown that decompressive craniectomy reduces mortality; however, functional outcomes remain variable, and optimal prognostic markers are still lacking [3]. Previous observational studies have reported mortality rates ranging from 40% to 70% following decompressive surgery, particularly in elderly patients and those with severe neurological impairment [11]. Consistent with these findings, our study demonstrated a high overall in-hospital mortality rate of 61.3%, emphasizing the need for improved risk stratification strategies.

Age has long been recognized as one of the most important predictors of outcome after large hemispheric infarction and decompressive craniectomy [12]. Advanced age is associated with reduced neuroplasticity, increased comorbidity burden, impaired autoregulatory mechanisms, and exaggerated inflammatory responses following ischemic injury [13]. In our cohort, older patients not only had significantly higher mortality but also exhibited increased SII levels, suggesting a potential link between age-related immune dysregulation and worse clinical outcomes.

Inflammation plays a central role in the pathophysiology of ischemic brain injury. Experimental and clinical studies have shown that neutrophil infiltration contributes to blood–brain barrier disruption, cerebral edema formation, and secondary neuronal damage [5,6]. Accordingly, peripheral inflammatory biomarkers have emerged as valuable prognostic tools in stroke populations. Elevated NLR has been associated with larger infarct volume, early neurological deterioration, and increased mortality in acute ischemic stroke [7,8,14]. Our results corroborate these observations by demonstrating significantly higher NLR values among non-survivors and identifying NLR as an independent predictor of mortality in multivariate analysis.

More recently, the systemic immune–inflammation index has been proposed as a more comprehensive indicator of thrombo-inflammatory activity by integrating neutrophil, lymphocyte, and platelet counts [9]. Several studies have reported that SII is associated with poor functional outcomes, hemorrhagic transformation, and mortality in patients with ischemic stroke [10,15,16]. In our study, SII demonstrated the highest predictive performance among inflammatory markers in ROC analysis, with an AUC of 0.833 and optimal sensitivity and specificity values. Moreover, SII remained an independent predictor of mortality after adjustment for confounding variables, suggesting that it may provide incremental prognostic value beyond conventional markers such as NLR.

The association between elevated SII and increased mortality may be explained by multiple mechanisms. Platelets play a crucial role in thrombus propagation and microvascular occlusion, while neutrophil-mediated inflammatory cascades exacerbate cerebral edema and oxidative stress [17]. Conversely, reduced lymphocyte counts reflect impaired adaptive immune regulation and increased vulnerability to post-stroke infections and systemic complications [18]. Therefore, SII may better capture the overall inflammatory and prothrombotic milieu that contributes to unfavorable outcomes following malignant infarction.

Another important finding of our study was the independent association between elevated serum creatinine and mortality. Renal dysfunction has been previously linked to worse outcomes in stroke patients, possibly due to endothelial dysfunction, chronic inflammation, and impaired hemodynamic stability [19]. Similarly, hypertension was identified as an independent predictor of mortality, consistent with prior reports suggesting that chronic vascular remodeling and reduced cerebrovascular reserve may worsen cerebral edema dynamics and perioperative complications [20].

Preoperative anticoagulant therapy also emerged as a significant predictor of mortality in our cohort. Although anticoagulation is commonly used in patients with cardioembolic stroke, it may increase the risk of hemorrhagic transformation and complicate perioperative management [21]. Previous studies have demonstrated that anticoagulant use is associated with higher rates of postoperative bleeding and worse functional outcomes after neurosurgical interventions [22].

Advanced age was significantly associated with increased in-hospital mortality in our cohort. Patients aged ≥ 65 years demonstrated substantially higher mortality rates than younger patients, consistent with previous studies showing poorer outcomes following decompressive craniectomy in elderly individuals [23]. Advanced age may contribute to worse prognosis through reduced neuroplasticity, a greater burden of comorbidities, and diminished physiological reserve. Nevertheless, recent evidence suggests that carefully selected older patients may still benefit from surgical intervention, highlighting the importance of individualized treatment decisions [24].

The present study has several strengths. First, we evaluated both traditional and novel inflammatory biomarkers in a relatively homogeneous cohort of patients undergoing standardized surgical treatment. Second, we performed comprehensive statistical analyses including ROC analysis and multivariable regression modeling to identify potential predictors of mortality. Finally, our findings provide exploratory cut-off values for SII that may facilitate early risk stratification in emergency settings. However, these thresholds were derived from a relatively small retrospective cohort and should be considered hypothesis-generating until validated in larger independent populations.

Nevertheless, certain limitations should be acknowledged. The single-center retrospective design may limit the external validity and generalizability of our findings to other institutions and patient populations. In addition, the exclusion of patients with incomplete laboratory data and other predefined exclusion criteria may have introduced selection bias, potentially affecting the representativeness of the study cohort. Furthermore, the study period extended from 2014 to 2024, during which substantial advances occurred in acute ischemic stroke management, including wider adoption of mechanical thrombectomy, evolving patient selection criteria, and improvements in neurocritical care. These temporal changes may have influenced patient outcomes and introduced residual confounding that could not be fully accounted for in this retrospective analysis. Additionally, inflammatory markers were measured only at admission, and dynamic changes during the postoperative period were not evaluated. Long-term functional outcomes were also not assessed. This limitation is particularly relevant in patients with malignant MCA infarction, in whom survival alone does not fully reflect treatment success. Following decompressive craniectomy, many survivors may experience substantial neurological disability, and functional outcome measures such as the modified Rankin Scale (mRS), Glasgow Outcome Scale (GOS), or Glasgow Outcome Scale–Extended (GOSE) are often considered equally important as mortality. Therefore, although our findings demonstrate an association between SII and in-hospital mortality, the relationship between inflammatory burden and long-term neurological recovery remains uncertain. Future studies should incorporate standardized functional outcome assessments to determine whether SII predicts not only survival but also the quality of neurological recovery after decompressive craniectomy. An additional limitation relates to the relatively small sample size and the limited number of outcome events. Although variables with *p* < 0.10 in univariate analyses were entered into the multivariable logistic regression model, the number of deaths was relatively low compared with the number of covariates included. Therefore, the possibility of model overfitting and instability of odds-ratio estimates cannot be completely excluded. Consequently, the multivariable findings should be interpreted cautiously and considered hypothesis-generating until validated in larger prospective cohorts. Furthermore, several established prognostic factors in malignant MCA infarction, including infarct volume, dominant hemisphere involvement, exact timing of decompressive craniectomy, and reperfusion success after recanalization therapies, could not be comprehensively evaluated because these data were not consistently available in this retrospective cohort. Their omission may have resulted in residual confounding and should be addressed in future prospective investigations.

In conclusion, our study demonstrates that systemic inflammatory burden, particularly elevated SII, is strongly associated with increased in-hospital mortality in patients undergoing decompressive craniectomy for malignant MCA infarction. Integration of SII into preoperative risk assessment may help identify patients at high risk of in-hospital mortality, facilitate more informed discussions with families regarding prognosis, support perioperative monitoring strategies, and assist clinicians in tailoring postoperative intensive care management.

## Figures and Tables

**Table 1:** Baseline characteristics according to age groups in patients undergoing decompressive craniectomy for malignant middle cerebral artery infarction.

Variable	<65 Years (n = 10)	≥65 Years (n = 21)	*p* Value
**Demographic characteristics**			
Age *, years	**54.2 ± 7.1**	**72.8 ± 5.3**	**<0.001**
Female sex	4 (40.0)	11 (52.4)	0.48
**Clinical characteristics**			
Glasgow Coma Scale score	7.3 ± 2.8	6.1 ± 2.5	0.19
GCS < 8	5 (50.0)	15 (71.4)	0.23
Midline shift, mm	10.0 ± 4.1	11.6 ± 5.6	0.41
Midline shift ≥ 10 mm	5 (50.0)	14 (66.7)	0.36
Anisocoria at admission	2 (20.0)	7 (33.3)	0.43
Right-sided MCA infarction	6 (60.0)	13 (61.9)	0.91
**Laboratory findings**			
Hemoglobin	12.0 ± 2.2	12.9 ± 2.6	0.44
Serum glucose	130 ± 38	148 ± 50	0.30
Serum creatinine	0.92 ± 0.30	1.21 ± 0.46	0.07
Neutrophil count	11.0 ± 3.7	12.8 ± 4.3	0.25
Lymphocyte count	1.34 ± 0.60	1.13 ± 0.51	0.33
Platelet count	230 ± 60	245 ± 75	0.61
Neutrophil-to-lymphocyte ratio	9.6 ± 5.1	14.1 ± 7.0	0.07
Systemic immune–inflammation index *	**1900 ± 900**	**3300 ± 1850**	**0.002**
**Comorbidities**			
Hypertension	3 (30.0)	13 (61.9)	0.09
Diabetes mellitus	2 (20.0)	7 (33.3)	0.43
Atrial fibrillation	1 (10.0)	5 (23.8)	0.34
Coronary artery disease	1 (10.0)	4 (19.0)	0.52
Hyperlipidemia	2 (20.0)	6 (28.6)	0.60
Previous stroke	1 (10.0)	3 (14.3)	0.74
**Treatment characteristics**			
Time to surgery ≥ 24 h	4 (40.0)	13 (61.9)	0.24
Mechanical thrombectomy	2 (20.0)	2 (9.5)	0.39
Intravenous thrombolysis (tPA)	2 (20.0)	4 (19.0)	0.94
Anticoagulant therapy	2 (20.0)	10 (47.6)	0.12
**Radiological characteristics**			
Hemorrhagic transformation	5 (50.0)	14 (66.7)	0.36
**Outcome**			
In-hospital mortality *	**3 (30.0)**	**16 (76.0)**	**0.003**

* Statistically significant (*p* < 0.05). Values are expressed as mean ± standard deviation or number (percentage). Continuous variables were compared using Student’s *t*-test or Mann–Whitney U test, and categorical variables using χ^2^ or Fisher’s exact test. Bold values indicate statistically significant results; bold row headings indicate table subcategories.

**Table 2:** Predictors of in-hospital mortality in patients undergoing decompressive craniectomy for malignant middle cerebral artery infarction.

Variable	Deceased (n = 19)	Survivors (n = 12)	*p* Value
**Demographic characteristics**			
Age *, years	**71.8 ± 8.9**	**58.6 ± 11.2**	**0.002**
**Clinical characteristics**			
Preoperative Glasgow Coma Scale score	5.7 ± 2.2	7.6 ± 3.2	0.067
Midline shift, mm	12.3 ± 5.7	9.2 ± 4.3	0.120
Time to surgery ≥ 24 h	13 (68.4)	4 (33.3)	0.06
Anisocoria	7 (36.8)	2 (16.7)	0.23
**Laboratory findings**			
Hemoglobin	13.2 ± 2.6	11.8 ± 2.1	0.121
Serum glucose	156 ± 48	126 ± 36	0.08
Serum creatinine *	**1.26 ± 0.47**	**0.92 ± 0.28**	**0.03**
Neutrophil count *	**13.5 ± 4.0**	**10.0 ± 3.3**	**0.018**
Lymphocyte count *	**1.01 ± 0.40**	**1.51 ± 0.67**	**0.033**
Platelet count	254 ± 74	218 ± 61	0.169
**Inflammatory markers**			
Neutrophil-to-lymphocyte ratio *	**15.0 ± 6.2**	**8.9 ± 6.1**	**0.013**
Systemic immune–inflammation index *	**3720 ± 1640**	**1830 ± 1350**	**0.002**
**Comorbidities**			
Hypertension *	**13 (68.4)**	**3 (25.0)**	**0.02**
Diabetes mellitus	7 (36.8)	2 (16.7)	0.25
Atrial fibrillation	5 (26.3)	1 (8.3)	0.22
**Treatment characteristics**			
Anticoagulant therapy *	**10 (52.6)**	**2 (16.7)**	**0.04**
**Radiological characteristics**			
Hemorrhagic transformation	14 (73.7)	5 (41.7)	0.08

* Statistically significant (*p* < 0.05). Values are expressed as mean ± standard deviation or number (percentage). Continuous variables were compared using Student’s *t*-test or Mann–Whitney U test, and categorical variables using χ^2^ or Fisher’s exact test. Bold values indicate statistically significant results; bold row headings indicate table subcategories.

**Table 3:** Receiver operating characteristic (ROC) analysis of laboratory and inflammatory markers for predicting in-hospital mortality.

Variable	AUC (95% CI)	Cut-Off	Sensitivity (%)	Specificity (%)	*p* Value
Hemoglobin	0.664 (0.46–0.86)	12.5	63	67	0.12
Neutrophil count *	**0.750 (0.57–0.93)**	**11.8**	**74**	**67**	**0.018**
Lymphocyte count *	**0.268 (0.08–0.46)**	**1.2**	**68**	**58**	**0.033**
Platelet count	0.601 (0.39–0.81)	230	58	58	0.16
Neutrophil-to-lymphocyte ratio *	**0.776 (0.60–0.95)**	**11.5**	**79**	**67**	**0.013**
Systemic immune–inflammation index *	**0.833 (0.67–0.99)**	**2850**	**84**	**75**	**0.002**

* Statistically significant (*p* < 0.05). AUC: Area under the curve; CI: Confidence interval. Optimal cut-off values were determined using the Youden index. Bold values indicate statistically significant results.

**Table 4:** Multivariate logistic regression analysis for independent predictors of in-hospital mortality.

Variable	Odds Ratio (OR)	95% Confidence Interval	*p* Value
Age (per year increase) *	**1.08**	**1.01–1.16**	**0.028**
Preoperative GCS score	0.81	0.62–1.05	0.11
Midline shift (per mm)	1.09	0.97–1.23	0.14
Serum creatinine *	**1.95**	**1.05–3.62**	**0.034**
Neutrophil-to-lymphocyte ratio *	**1.12**	**1.01–1.25**	**0.039**
Systemic immune–inflammation index (per 1000 units) *	**1.41**	**1.10–1.82**	**0.006**
Hypertension *	**2.76**	**1.01–7.54**	**0.048**
Anticoagulant therapy *	**3.18**	**1.05–9.61**	**0.041**

* Statistically significant (*p* < 0.05). Variables with *p* < 0.10 in univariate analysis were entered into the multivariate logistic regression model. Results are presented as odds ratios (ORs) with 95% confidence intervals (CIs). Bold values indicate statistically significant results.

## Data Availability

The data supporting the findings of this study are available from the corresponding author upon reasonable request. The data are not publicly available due to privacy and ethical restrictions involving patient information.
